# Being the Ghost in the Machine: A Medical Ghostwriter's Personal View

**DOI:** 10.1371/journal.pmed.1001071

**Published:** 2011-08-09

**Authors:** Linda Logdberg

**Affiliations:** Biologist, Fernbank Science Center, Atlanta, Georgia, United States of America

## Abstract

Linda Logdberg, a medical ghostwriter for 11 years, provides a personal view of her former work and what she believes should be done about the problem of fraud in authorship.

## Introduction

Ethical concerns about medical ghostwriting have been directed primarily at “guest” authors and the pharmaceutical companies that pay them. One voice that is largely missing is that of the ghostwriters themselves who, after all, create the documents that are in the ethical and legal crosshairs. Without them, one could argue, there can be no fraud, because it is they who create the fraudulent product.

For almost 11 years, I worked as a medical writer, creating a variety of pieces including the occasional ghostwritten article. For the most part, I never saw the finished paper, nor did I care to. This article describes what I did, why I did it, why I stopped doing it, and what I think might be done about the problem of fraud in authorship.

## What I Did

In line with the description on the American Medical Writers' Association Web site about what medical writers do [1], I wrote slide kits, monographs, executive summaries, journal articles, backgrounders, newsletters, competitive analyses, publication plans, video scripts, audio scripts, and continuing medical education (CME) programs for physicians and nurses. Each piece (“job”, in advertisingspeak) was born out of the publications planning strategy developed for a fee by the medical education (meded) company for the pharmaceutical corporation.

Medical writers are highly deadline-driven. For one hormone patch product I worked on, writers and “creatives” were asked to remain at work until close to midnight to await results from physician focus groups on the West Coast. After receiving the client's (i.e., pharma's) take on the focus group results for that day, we rewrote the messages for the next day's groups and sent them to the West Coast. A slide rose or fell on subtleties: in one slide kit draft in my files, an account executive added “Importance of early intervention” to a slide titled “Chronic Pain.” The bullet does not help define chronic pain, but it plants the idea that treatment should be started ASAP in the mind of the listener. Clients admonished us to always distinguish between “adverse effects” (for competitors' products: Drug X could have *caused* the heart attack) and adverse events (our product: some patients taking Drug X *just happened to have* a heart attack).

Ghostwriting was a small, but real, part of my duties. I have seen published pieces that are virtually identical to the final drafts I submitted. Regardless of what I wrote, though, for many years I considered my role to be similar to that of a highly paid technician and did not question its ethics.

## Why I Did It

My background may not have been typical for a medical writer, but neither was it uncommon. I enjoyed a research career up to the point where I no longer enjoyed it, which came a few years after receiving my PhD. Several things about an academic career did not encourage me to continue, although I loved research and working in the lab. These included the difficulty of getting tenure and the possibility of finding myself unemployed in my mid-40s: there were 12 newly hired assistant professors in the department where I did my second postdoc, with an average time to tenure of more than 10 years.

Ironically, though, it was the ethics of authorship that sent me fleeing academia. I ran afoul of a colleague in my last research position, who assumed that postdocs would draft his grant renewal application. I commented offhandedly one day, “Well, I for one would never write something and have someone else sign his name to it—that would be *unethical*.” Dr. X told me that that was when he realized that it would not work out for me to continue there, as my attitude was unacceptably insubordinate. Faced with the need for a job, I resigned and answered an ad in *The New York Times* for a company that needed medical writers. This began a series of freelance and in-house jobs with a range of medical communications companies.

I believe that many of the factors that kept me in medical writing apply to most medical writers. First, I believed that I was helping people: sick people need drugs, and physicians need to know about those drugs to prescribe them appropriately. Second, I had young children and valued the flexibility of working at home, which most meded companies offered at least part of the time. Third, the work was interesting: I interacted with top researchers and was assured of an ease of access that I never would have had as an assistant professor. Fourth, the money was good. *Really* good, especially compared with the typical assistant professor salary. And perhaps most important in the longer run—it was fun. Traveling, eating in high-end restaurants, wearing fashionable clothes, and rushing to meet important deadlines—what's not to like?

## Why I Stopped Doing It

It turned out, there was quite a bit not to like. I'd started in smaller companies headed by PhDs or MD/PhDs who dealt directly with the primary researchers and the pharmaceutical companies. There were no advertising types in sight, and I had frequent, direct communication with the physician-authors. I saw my role as helping a busy researcher write up research results: he or she did the research (which I'd already decided I didn't want to do), and I got to analyze and describe it.

But as my career developed, several of these smaller firms went out of business, and I began to get more work from larger meded companies that were part of large advertising agencies. The bigger the agency, the more likely it was that my contact person was someone without a science background. In the worst of these settings, I discussed projects only with the program manager and had limited—or no—access to the “author.”

The work itself began to lose its charm. My preferred area of interest was oncology, and the lighter-weight assignments that increasingly came my way were not as interesting. It was hard to muster up much enthusiasm for the importance of treating, say, subclinical hypothyroidism—indeed, subclinical *anything*. In addition, the ethical issues began to tap me on the shoulder: perhaps the most memorable example of this was a contraceptive product that caused severe, unpredictable vaginal bleeding in some women. My job was to draft a monograph that would profile the product's benefits, one of which, according to the client, was that although the bleeding could be severe, it was at least something that women could *anticipate*. In other words—the bad news is that a meteorite will strike you, but the good news is—a meteorite will strike you!

This kind of doublespeak became more and more troubling, and my career came to an end over a job involving revising a manuscript supporting the use of a drug for attention deficit-hyperactivity disorder (ADHD), with a duration of action that fell between that of shorter- and longer-acting formulations. However, I have two children with ADHD, and I failed to see the benefit of a drug that would wear off right at suppertime, rather than a few hours before or a few hours after. Suppertime is a time in ADHD households when tempers and homework arguments are often at their worst. So I questioned the account executive at the large agency that had hired me. In particular, I wanted to ask the physician author their view of the drug's benefits. Attempts to discuss my misgivings with the meded contact met with the curt admonition to “just write it.” But perhaps because this particular disorder was so close to home, I was unwilling to turn this ugly duckling of a “me-too” drug into a marketable swan.

I decided it was time to burn my medical writing bridges and contacted *The New York Times*, which coincidentally had planned an investigative article on pharmaceutical marketing to physicians. I was interviewed for this article, written by Melody Petersen, by Ms. Petersen and Walt Bogdanich [2]. Shortly after its publication (November 22, 2002; page A1), I received a polite letter from an executive of the meded company asking for all the materials back and reminding me of my confidentiality agreement. I also received a direct threat of legal retaliation in a phone call from my former contact at that agency.

## What I Think Now

Wordsmithing is ubiquitous in all promotional writing, not just ghostwriting: it's the name of the game. Yet advertising masquerading as unbiased health information clearly threatens the fundamental assumptions of scientific research. Can pharma, clinicians, researchers, and consumer protection advocates work together without distortion?

I believe that they can. A system could be put in place that fortuitously addresses another critical problem—the underemployment of medical writers, who, possessing academic training and experience without opportunities to use them, are “all dressed up” intellectually with no place to go. All too often, people like me find themselves unemployed or in science-related positions such as teaching that offer little hope of advancement in a job market that has not added new jobs in biomedicine in 20 years despite a doubling in the number of PhDs in that field [3].

If research centers that employ people who serve as “guest authors” (often the same places that accredit CME programs funded by pharmaceutical money) were, in addition, to employ medical writers, much could be accomplished toward cleaning up the ethics of authorship. Funds to pay medical writers and editors could be given to these centers by pharmaceutical companies, allowing the writers to work directly with researchers. The pharmaceutical company's role would be limited to factchecking the document and clarifying issues about dosage, adverse events, postmarketing developments, etc., and the final product would be submitted for peer review by the researcher personally. The incentive for the pharmaceutical company would be to educate and inform physicians and researchers, pure and simple. Drug promotion would still occur, but would be in the hands of advertising agencies.

This approach would eliminate the meded companies, currently “the middleman” between pharma and physician. It would reduce the need for journals to take on the entire responsibility of vetting submitted manuscripts for conflicts of interest related to authorship, because the academic institution that employed the researcher-author would have a stake in ensuring the paper's accuracy as well as in exposing conflicts of interest. The increased visibility to the research community of the pharmaceutical company could reduce the likelihood of unfounded claims or egregious promotion of off-label use. This arrangement could shorten the interval between research and publication, and ensure a high quality of publications. Finally, one other stakeholder would surely be well pleased by such an arrangement—the medical writer, who would be glad to once again work in an academic environment.

## Abbreviations

ADHD: attention deficit-hyperactivity disorder
CME: continuing medical education
meded: medical education
